# Is Vancomycine Still a Choice for Chronic Osteomyelitis Empirical Therapy in Iran?

**DOI:** 10.5812/ircmj.2165

**Published:** 2012-12-06

**Authors:** Morteza Izadi, Mohammad Mahdi Zamani, Seyed Ahmad Mousavi, Seyed Mir Mostafa Sadat, Zeinab Siami, Noushin Vais Ahmadi, Nematollah Jonaidi Jafari, Shahram Shirvani, Mojgan Majidi Fard, Abbas Ali Imani Fooladi

**Affiliations:** 1 Health Research Center (HRC), Baqiyatallah University of Medical Sciences, Tehran, IR Iran; 2 Orthopedics Department, Medicine faculty, Tehran University of Medical Sciences, Tehran, IR Iran; 3 Subspeciality Clinic of Osteomyelitis, Sasan Hospital, Tehran, IR Iran; 4 Applied Microbiology Research Center, Baqiyatallah University Medical of Sciences, Tehran, IR Iran

**Keywords:** Iran, Methicillin-Resistant, Osteomyelitis, Staphylococcus Aurous, Vancomycin Resistance

## Abstract

**Background:**

Pyogenic bacteria and especially *Staphylococcus aurous* (*S. aurous*) are the most common cause of chronic osteomyelitis. Not only treatment protocol of chronic osteomyelitis occasionally is amiss but also this malady responds to treatment difficultly.

**Objectives:**

This study investigates antibiotic resistance pattern of *S. aurous* isolated from Iranian patients who suffer from chronic osteomyelitis by two methods: disk diffusion (Kirby bauyer) and E-test (Epsilometer test) to find Vancomycin susceptibility and MIC (Minimum inhibitory concentration).

**Patients and Methods:**

One hundred and thirty one patients who suffer from chronic osteomyelitis which have been referred to both governmental and private hospitals at 2010 were tried out for culturing of osteomyelitis site (sites). Antibiotic susceptibility and MIC of isolated bacteria were investigated by Kirby bauyer and E-test respectively.

**Results:**

Samples were collected from bone (73.4%), surrounding tissue (14.6%) and wound discharge (12%). *S. aureus* was isolated from 49.6% of the samples. According to disc diffusion, methicillin resistance *S. aureus* (MRSA) was 75% and Vancomycin resistance *S. aurous* (VRSA) was 0% and based on MIC, MRSA was 68.5% and VRSA was 0%. According to MIC experiments, maximum sensitivity was against to Vancomycin (90.2%) and ciprofloxacin (54.4%) respectively but based on disc diffusion, maximum sensitivity was against to Vancomycin (97.7%) and ciprofloxacin (43.2%), respectively (*P* = 0.001). E-test (9.8%) in comparison with Disc diffusion (2.3%) showed higher percent of intermediate susceptibility to Vancomycin (*P* = 0.017).

**Conclusions:**

Comparison of antibiograms and MICs showed that Kirby bauyer technique especially for detection of VISA strains is not reliable comparison with E-test. Already VRSA strains have not detected in Iranian chronic osteomyelitis, Thus Vancomycin is the first choice for chronic osteomyelitis empirical therapy in Iran yet.

## 1. Background

Osteomyelitis is an invasive and hard-treatable infectious disease conducted to periosteum and cortex of osteon by aversion canals and it will cause necrosis if periosteum is infected. Recurrent exacerbations in chronic osteomyelitis cause to non-responding to short period treatment (1). Presence of collagen leads to microorganism adhesion to osteon’s matrix (2). Various pathogens play a role in chronic osteomyelitis which depend on several factors such as age and medical history. Adults are usually infected by *S. aurous* and gram negative bacteria (3). There is a variety in the organisms of chronic osteomyelitis in different regions across the globe. In most European and North American studies, the most common bone infections have been reported, are Staphylococci and Streptococci with Neisseria gonorrhea, particularly prevalent in North America (4).

More expenses and poor prognosis would be expected when appropriate antibiotic treatment is delayed (5, 6). While the responsible organism is not definite, the first choice of antibiotics is empirical. *S. aureus*, as a main agent of chronic osteomyelitis (7), attaches to osteon’s matrix receptors and affects fibronectin, laminin and cialo glycoproteins of matrix (8). Staphylococcus includes 30 species, at least three of which have clinical symptoms as osteomyelitis: *S. aurous, S. epidermidis, S. Saprophyticus* (7). Considerable controversy still exists over the current and future roles of Vancomycin in controlling serious Methicillin-Resistant Staphylococcus Aurous (MRSA) infections. Therefore, periodic studies on Vancomycin resistance *S. aurous* (VRSA) and Vancomycin-intermediate *S. aurous* (VISA) are substantial (9). *S. aurous* strains have reduction teicoplanin susceptibility without a clearly demonstrated reduction in Vancomycin susceptibility (10), while VISA strains have demonstrated reduced teicoplanin susceptibility (11). Nowadays, however, focusing on VISA studies for preventing resistance strains is more valuable compared to VRSA ones.

On the other hand, chronic osteomyelitis does have a heavy burden on affected patients and societies. Several orthopedic surgeries and hospitalizations are needed, thus vast expenses of treatment are necessitated in chronic osteomyelitis sufferer; these factors make low compliance and consequently low sufferers' consent to antibiotic therapy entirely. Especially in developing countries, excessive antibiotic consuming in these patients yield to *S. aurous* multidrug resistance strains (12) and generally periodic surveillances on *S. aurous* as the main etiology of this expensive malady is necessitated to provide appropriate empirical treatment of infections circulating in every geographic region (13). In previous studies epsilometer test (E-test) comparing with Disc diffusion especially on brain heart infusion agar plate has shown no error in resistant and intermediate susceptibility to Vancomycin for Enterococcus (14), also about another organism such as Haemophilus influenza, E-test is reliable for resistant and intermediate antibiotic susceptibility in comparison with Disc diffusion (15).

## 2. Objectives

This study investigates antibiotic resistance pattern of *S. aureus* isolated from patients who suffered from chronic osteomyelitis by two laboratory methods (Kirby bauyer and E-test).

## 3. Patients and Methods

### 3.1. Subjects

131 patients with osteomyelitis continued for 6 months at least, entered our cross-sectional study. Subjects had been referred to two referral hospitals of Tehran, Iran (one teaching hospital and one private hospital) during the year 2010. None of the subjects had received any antibiotics during 2 last weeks before entering the study. Subjects who had to receive antibiotics due to their critical conditions, were excluded from the study.

### 3.2. Sampling

All subjects were visited by both infectious disease and orthopedics specialists and site of biopsy was chosen by at least one of them. Bone biopsy, surrounding tissue and wound discharge sampling were done by orthopedic specialist at operation room under sterile condition. Samples were transferred to laboratory by transport medium straightaway.

### 3.3. Laboratory Analysis

All samples were subcultured onto Mueller Hinton agar plates and incubated at 37°C for 24 hours. A subculture of each strain was suspended in 2 ml of nutrient broth and then adjusted to a turbidity equivalent of 0.5 McFarland. 1 µl of each inoculum was sub cultured on Mueller Hinton agar plates monotonously. In case of negative culture result of bone biopsy, surrounding tissue culture result was considered and if surrounding tissue culture result was negative, wound discharge culture result was counted as patient’s culture result. The E-test strips and disc of antibiotics placed on distinct plates. Disc of Clindamycin, Trimethoprim-sulfamethoxazole, Ciprofloxacin, Tetracycline, Oxacillin, Vancomycin, Penicillin, Amoxicillin, Cephalexin, Doxycyclin, Erythromycin were posed on plates for disc diffusion test. Strips of Oxacillin, Vancomycin, Clindamycin, Trimethoprim-sulfamethoxazole, Tetracycline and Ciprofloxacin were placed for E-test. The diameters of inhibitory zones were obtained by disk diffusion test and MIC (Minimum inhibitory concentration) results were obtained by E-test method after 24 hours of incubation at 35°C. In the disk diffusion test, diameters of inhibitory zones were calculated and results were evaluated according to the interpretive criteria of CLSI (Clinical and laboratory standards institute), 2006. For the E-test, elliptical zone of inhibited growth were calculated and three interpretive categories of susceptibility results (sensitive, intermediate, and resistant) were defined according to company manual. All mentioned substances had been provided by a domestic manufacturer (Padtan Company).

### 3.4. Statistical Analysis

Statistical analyses were done using the chi-square test with statistical package of social sciences, version 16.0 (SPSS Inc, Chicago, IL). A *P* value of less than 0.05 was considered significant.

## 4. Results

**Figure 1:**
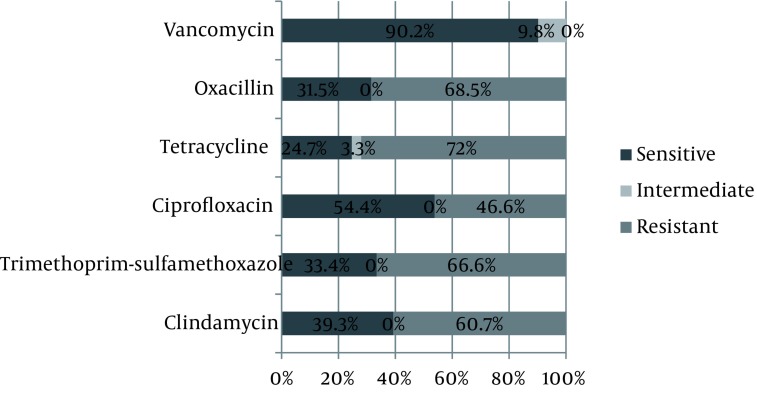
Growth Species From Osteomyelitis Tissue Biopsies

**Figure 2:**
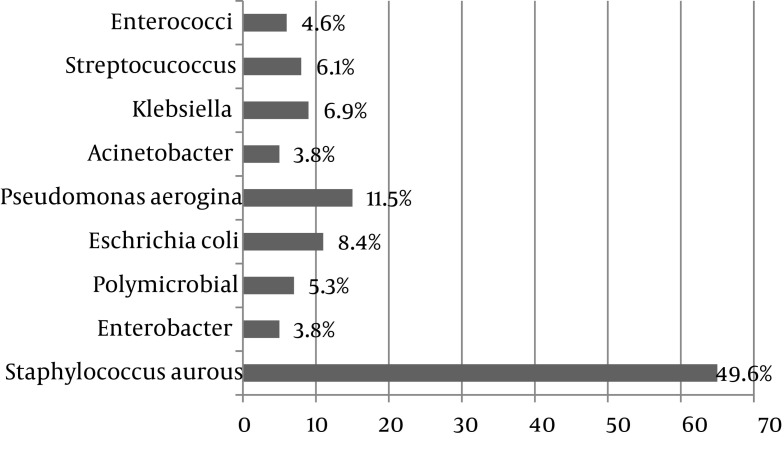
Antibiotic Susceptibility for *S. aurous* by Disc Diffusion

**Figure 3:**
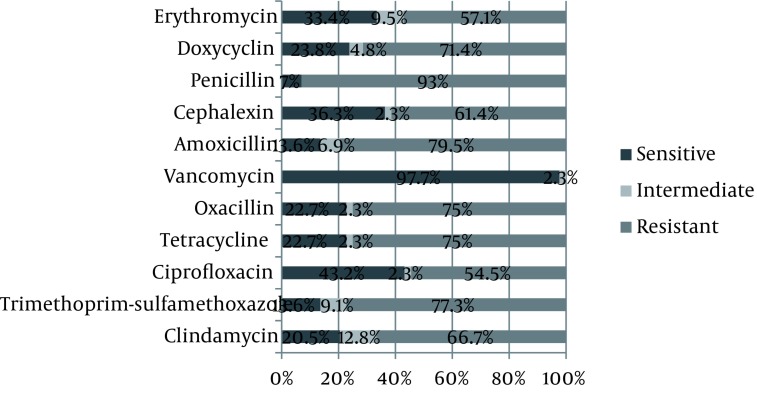
Antibiotic Susceptibility for *S. aurous* by E-Test

107 male (81.7%) and 24 female (18.3%) subjects with chronic osteomyelitis were included in this study (male to female ratio was 4.4:1). Mean age was 42.59 years with age range of 10 to 67 years. The most common affected bones in these patients were Tibia (33%) and Femur (27%), followed by Hip (14%), Foot (14%), Humorous (7%) and Malleolus (5%). The most positive subcultures were obtained from bone (73.4%) and the least ones were taken from wound discharge (12%), while 14.6 percent of positive subcultures were taken from Surrounding tissue. The most common species grown from samples was *S. aurous* (48.9%). Growth species were categorized in Figure 1. *S. aurous* had the most sensitivity to Vancomycin (97.7%) and the least sensitivity to Penicillin (7%). Figure 2 summarizes the comparative results of disc diffusion. Antibiotic susceptibility patterns regarding E-test are shown in Figure 3. *S. aurous* had the most sensitivity to Vancomycin (90.2%) and the lowest to Tetracyclin (24.7%). MRSA pertain to disc diffusion and E-test was 75% and 68.5% respectively and VRSA calculated zero percent regarding both methods. VISA counted 2.3% by disc diffusion and 9.8% by E-test. E-test showed higher percent of intermediate susceptibility to Vancomycin in comparison with Disc diffusion (*P* = 0.017).

## 5. Discussion

Chronic osteomyelitis has a heavy burden on the affected patients and societies. Several orthopedic surgeries and hospitalizations are needed, however, vast expenses of treatment are necessitated in chronic osteomyelitis sufferer (16). In many parts of the world, including Iran, there are not enough studies on the exact scale of causative organisms of osteomyelitis. To our knowledge, in Iran as a representative of Middle East, Only two studies investigated osteomyelitis characteristics and causative organisms (17, 18). In the first study, Kalantari *et al*. analyzed 145 cases of septic arthritis and osteomyelitis in 2007 where 84 cases proved to have osteomyelitis. Age range was 6 days to 15 years with male to female ratio of 1.2:1. Overall, *S. aurous* was recovered from 61% of positive cultures and was the most frequent organism which grew. VISA by disc diffusion (11%) was reported more than our study (2.3%); however, 48% of osteomyelitis cases had arthritis simultaneously. Femur and tibia with 13.7% and 11%, respectively, were the most common bones involved with osteomyelitis, this finding is the same as our study; nevertheless, rate of involvement in these bones was higher in our study. The probable reason is pure osteomyelitis in our survey versus companionship of osteomyelitis and arthritis in about half of cases in that study. Knee, hip, and ankle consistently account for at least 80 percent of pediatric arthritis (19).

Second study was done on war handicapped chronic osteomyelitis by authors of this paper; in that retrospective study, *S. aureus* was ranked first among causes of osteomyelitis (51.9%) in war handicapped chronic osteomyelitis where all subjects were male (17). Like the present study, the most common involving bones were femur (45%) and tibia (25%) in handicapped chronic osteomyelitis; We have observed almost the same result. *S. aurous* was the most positive result culture from chronic osteomyelitis (49.6%) among 131 subjects from general population compared to 40 subjects who were war handicapped (51.9%). This similar result can support similar protocol of treatment for war handicapped chronic osteomyelitis and general population patients.

There are several regional studies on *S. aurous* isolated from several infection sites except osteomyelitis. Mostafavizadeh *et al*. reported 75% MRSA strains from nosocomial infections by E-test which is different from our MRSA isolation rate. The probable reason for this can be the difference in location of samples (osteomyelitis versus nosocomial infections) and consequently difference in strains (20); Moreover, this confirms difference in *S. aurous* strains from different locations so if we are aware of MRSA and VISA and VRSA strains in nosocomial infections of Iran, It is needed to carry out similar periodical studies on osteomyelitis or other infection sites.

Kim *et al*. performed similar study in Koreans' chronic osteomyelitis. 49 patients with age range of 11 to 79 years and mean age of 47.3 years were examined for efficacy of surgery and antibiotic therapy with a new protocol. The others, however, did not report grown microorganisms details. Male to female ratio was 1.7:1 in their study (21). Demographic data was almost the same as our study but they had a wider age range.

In another study, Orimolade *et al*. investigated clinicopathological characteristics of Nigerian patients with acute and chronic (76.7%) osteomyelitis. In their study, male to female ratio was 2:1 and age range was above 15 years. *S. aureus* grew in 80.8% of grown samples (22) Which was more than our finding (49.6%). This higher result might be due to the presence of acute osteomyelitis among their participants. *Eschrichia coli* (7.7%), Pseudomonas aero Gina (3.8%) and Klebsiella (3.8%) were the most common germ after *S. aurous* in Nigerian osteomyelitis, similar to our study, with a difference in ranks; In Iranian osteomyelitis, Pseudomonas aero Gina (11.5%), *E. coli* (8.4%) and Klebsiella (6.9%) were ranked after *S. aureus* respectively. In our study the most resistance of *S. aurous* was to Penicillin (93%) and Amoxicillin (79.5%) and Trimethoprim-sulfamethoxazole (77.3%) based on disc diffusion in Iranian osteomyelitis and the most sensitivity to Vancomycin (97.7%) ; Also based on E-test, the most sensitive antibiotic was Vancomycin (90.2%). This pattern underscores the importance of Vancomycin empirical therapy in Iranian osteomyelitis. MRSA was reported 75% by disc diffusion; though significantly, it was determined in a smaller scale by E-test (68.5%). Moreover, disc diffusion results showed 2.3% strains as VISA which was less than E-test results (9.8%) significantly. These results support E-test as a reliable test for osteomyelitis. On the other hand, 5-microgram disk diffusion test is easy to perform but does not give real results about VISA similar to E-test method; certainly, disk diffusion is substantialy enough to determine a clinical choice for antibiotic therapy in chronic osteomyelitis and our findings support that.

Overall, clinical trials of Iranian osteomyelitis are needed in future to evaluate exact results of E-test by clinical response to antibiotics. Although our study supported Vancomycin empirical therapy, detection of a reliable method for finding a right narrow spectrum antibiotic can induce lower empirical therapy and consequently keep VISA in a low level and prevent construction of VRSA strains in Iranian chronic osteomyelitis.

The attainment of antimicrobial resistance and changing patterns of staphylococcal disease have been common themes in the staphylococcal literature over the past 50 years and there is not any exception for osteomyelitis causative staphylococci. Therefore, periodic evaluation of VRSA and VISA should be performed in order to be prepared for VRSA strains and also in case of sudden increasing in level of VISA strains, by executing more studies on genotype, biological behavior and etc. of new kinds of VISA. There is no report of VRSA in Iranian osteomyelitis; However, according to previous reports of Iranian specimens, VRSA strains do exist in Iran, (23) although there is no report of VRSA in Iranian osteomyelitis already. So Imipenem and other carbapenems are not suggested especially in Iran.

There are no VRSA strains in Iranian osteomyelitis and thus Vancomycin empirical therapy for chronic osteomyelitis is choice for the present time and periodical studies, especially by E-test, can make us aware of VRSA strains' presence and VISA strains'. In case of these two events, serious response should be triggered to alter osteomyelitis treatment protocol.
